# Rapid outpatient transient ischemic attack clinic and stroke service activity during the SARS-CoV-2 pandemic: a multicenter time series analysis

**DOI:** 10.3389/fneur.2024.1351769

**Published:** 2024-02-07

**Authors:** Andy Lim, Peter M. Rothwell, Linxin Li, Shelagh B. Coutts, Michael D. Hill, Maria Guarino, Valentina Barone, Francesca Rondelli, Timothy Kleinig, Reid Cornell-Farrow, Martin Krause, Miriam Wronski, Shaloo Singhal, Henry Ma, Thanh G. Phan

**Affiliations:** ^1^School of Clinical Sciences at Monash Health, Monash University, Melbourne, VIC, Australia; ^2^Wolfson Center for the Prevention of Stroke and Dementia, Nuffield Department of Clinical Neuroscience, John Radcliffe Hospital, University of Oxford, Oxford, United Kingdom; ^3^Departments of Clinical Neurosciences, Radiology and Community Health Sciences, Hotchkiss Brain Institute, University of Calgary, Calgary, AB, Canada; ^4^IRCCS Istituto delle Scienze Neurologiche di Bologna, Bologna, Italy; ^5^Dipartimento di Scienze Biomediche e Neuromotorie, Università di Bologna, Bologna, Italy; ^6^Department of Neurology, Royal Adelaide Hospital, Adelaide, SA, Australia; ^7^Department of Neurology, Royal North Shore Hospital and Kolling Institute, University of Sydney, St Leonards, NSW, Australia; ^8^Department of Neurology, Monash Health, Melbourne, VIC, Australia

**Keywords:** stroke, TIA, TIA clinic, SARS-CoV-2, time series analysis

## Abstract

**Background and aim:**

Rapid outpatient evaluation and treatment of TIA in structured clinics have been shown to reduce stroke recurrence. It is unclear whether short-term downtrends in TIA incidence and admissions have had enduring impact on TIA clinic activity. This study aims to measure the impact of the pandemic on hospitals with rapid TIA clinics.

**Methods:**

Relevant services were identified by literature search and contacted. Three years of monthly data were requested – a baseline pre-COVID period (April 2018 to March 2020) and an intra-COVID period (April 2020 to March 2021). TIA presentations, ischemic stroke presentations, and reperfusion trends inclusive of IV thrombolysis (IVT) and endovascular thrombectomy (EVT) were recorded. Pandemic impact was measured with interrupted time series analysis, a segmented regression approach to test an effect of an intervention on a time-dependent outcome using a defined impact model.

**Results:**

Six centers provided data for a total of 6,231 TIA and 13,191 ischemic stroke presentations from Australia (52.1%), Canada (35.0%), Italy (7.6%), and England (5.4%). TIA clinic volumes remained constant during the pandemic (2.9, 95% CI –1.8 to 7.6, *p* = 0.24), as did ischemic stroke (2.9, 95% CI –7.8 to 1.9, *p* = 0.25), IVT (−14.3, 95% CI −36.7, 6.1, *p* < 0.01), and EVT (0, 95% CI –16.9 to 16.9, *p* = 0.98) counts. Proportion of ischemic strokes requiring IVT decreased from 13.2 to 11.4% (*p* < 0.05), but those requiring EVT did not change (16.0 to 16.7%, *p* = 0.33).

**Conclusion:**

This suggests that the pandemic has not had an enduring effect on TIA clinic or stroke service activity for these centers. Furthermore, the disproportionate decrease in IVT suggests that patients may be presenting outside the IVT window during the pandemic – delays in seeking treatment in this group could be the target for public health intervention.

## 1 Introduction

Rapid outpatient evaluation and treatment of TIA has been shown to reduce stroke occurrence (1–3). With a structured pathway offering urgent evaluation and immediate antiplatelet and secondary prevention therapies, stroke risk has been reduced from 10.3 to 2.1% (2). The SARS-CoV-2 pandemic however, declared on the 11th of March 2020 by the World Health Organisation (4), has caused disruption to health services worldwide. Reductions in incidence of TIA have been observed in primary care with a 16% decrease noted across 1,262 general practices in Germany during the pandemic (5). Similar reductions have been noted in the hospital setting, with a 51% decrease in TIA incidence in one tertiary stroke center (6), 63% decline in TIA admissions across five stroke centers in the US (7), and a lower proportion of stroke center admissions being due to TIA (8). It remains unclear whether these early changes reflect a short-term downtrend or a new persisting baseline, as longer-term intra-pandemic data to describe TIA trends remains lacking.

The present study therefore aims to utilize interrupted time series analysis to test the effect of the COVID-19 pandemic over 3 years on centers with rapid TIA clinics identified by literature search. TIA presentations, ischemic stroke presentations, and trends in reperfusion therapies including IV thrombolysis (IVT) and endovascular thrombectomy (EVT) are analyzed. The study provides follow-up data to a recent paper of 16 rapid TIA clinics that documented various pandemic adaptations: five had adopted a different vascular imaging strategy, 10 had switched to exclusive telehealth consultation, and all remained active (9). Six of these 16 hospitals agreed to submit 3 years of data, and the results are shared here.

## 2 Methods

### 2.1 Study design

A multicenter observational study of 3 years of monthly presentation data from six centers using time series analysis to measure the effect of the pandemic on TIA, ischemic stroke, IV thrombolysis (IVT), and endovascular thrombectomy (EVT) counts. This study was approved by the Monash Health Human Research Ethics Committee (RES-20-0000-915Q-72167).

### 2.2 Setting

The initial paper identified 16 clinics via a literature review of rapid TIA pathways in PubMed (9). Six of these centers agreed to provide 3 years of data. Three were from Australia, and one center each from England, Canada, and Italy, and these are listed in Table 1. The publication of each original rapid TIA pathway paper ranged from 2007 to 2019 (1, 2, 10–14). The pathways were primarily completed in the emergency department, with the exception of the primary care based OXVASC study (2), and the combined ED and primary care based Foothills Medical Center pathway (12). All accepted TIA referrals; Oxfordshire (2) also accepted minor stroke in addition to TIA. Monash Medical Center and John Radcliffe reported using ultrasound for vascular imaging pre-COVID (9) with the rest using CT angiography. All centers were utilizing CT angiography for vessel imaging during the COVID period (9). While each service used their own unique inclusion and exclusion criteria for the rapid TIA pathway, two went further by prioritizing time to review by mechanism (i.e., the presence of atrial fibrillation and/or >50% internal carotid artery stenosis) (1) or by timing of symptom onset and presence of other high-risk features (e.g., motor/speech deficit >5 min, ABCD2 ≥ 4, or atrial fibrillation) (12). All pathways commenced treatment immediately. The study duration included a baseline pre-COVID period (April 2018 to March 2020), and an intra-COVID period (April 2020 to March 2021), with the impact defined as the World Health Organization’s declaration of the pandemic on 11th March 2020 (15). Therefore, the first full intra-pandemic month was defined as April 2020. This changepoint was specified *a priori* to avoid exploratory analysis to discover a significant time point, as recommended by previous researchers (16). We did not include lockdown periods into the model given the heterogeneity in duration and intensity of stay-at-home orders. For example, Melbourne in Australia had multiple lockdowns, some only lasting for several days. We felt that this would not fit into a model of monthly counts.

**Table 1:** Characteristics of the included centers with rapid TIA clinics.

Service	Region	Country	Year of original TIA pathway publication	Initial diagnosis	Setting	Clinic inclusions:	Pre-COVID vascular imaging	Intra-COVID vascular imaging*	Rapid follow-up prioritized by:	Details of prioritization	Treatment timing
Monash Medical Center (1)	Victoria	Australia	2012	ED physician and vascular neurology trainee	ED based	TIA	US	CTA	Stroke mechanism	Presence of AF or > 50% ICA stenosis – as soon as possible Otherwise, 4–6 weeks	Started in ED
Royal North Shore (10)	Sydney	Australia	2016	ED physician and vascular neurology trainee	ED based	TIA	CTA/US	CTA/US	Nil	ABCD2 < 4 for first 12 months of study After this, all TIA (fully resolved symptoms) provided history is not unclear, and symptoms are suggestive of TIA	Started in ED
Royal Adelaide (11)	Adelaide	Australia	2018	ED physician	ED based	TIA	CTA	CTA	Nil	If no high-risk cardiac source (AF with no or subtherapeutic anticoagulation or mechanical valve replacements) or CTA evidence of ICA stenosis >50%; otherwise, TIA is admitted	Started in ED
John Radcliffe Hospital/OXVASC (2)	Oxfordshire	England	2007	Primary care physician	Primary care based: 2002–2004: by referral Post-2004: patient asked to present directly to clinic, no appointment necessary	TIA and minor stroke	US	CTA	Clinical judgement	If the primary care physician thought the patient did not require immediate hospital admission	2002–2004: via primary care physician after treatment recommended by fax. Post-2004: immediately in clinic.
Foothills Medical Center (12)	Alberta	Canada	2012	ED or primary care physician	ED and primary care based, with access to a TIA triaging algorithm and TIA hotline	TIA	CTA	CTA	Timing and symptoms	High risk: Symptom onset <48 h with one of: Motor deficit lasting >5 min Speech deficit lasting >5 min ABCD2 ≥ 4 AF Medium risk: Symptom onset 48 h-7 days with one of: Motor deficit lasting >5 min Speech deficit lasting >5 min ABCD2 ≥ 4 Low risk: Symptom onset >7 days or < 7 days and no high-risk symptoms	High: 24 h Medium: 3 days Low: 2 weeks
Bologna (13)	Bologna	Italy	2015	ED physician and vascular neurologist	ED based	TIA	CTA/US	CTA/US	Timing, mechanism, and symptoms	If no high-risk features: >1 h duration Recurrent TIA within the previous 72 h New cardioembolic origin Carotid surgery need Intracranial symptoms stenosis ABCD2 ≥ 4 Need for monitoring for severe co-morbidities	Started in ED

*As at 2020 (9). ED, emergency department; TIA, transient ischemic attack; US, ultrasound; CTA, computed tomography angiography; OXVASC, Oxford Vascular Study; NIHSS, National Institutes of Health Stroke Scale.

### 2.3 Participants and variables

Patients presenting from April 2018 to March 2021 with ischemic stroke or TIA were included. TIA presentations were recorded either as clinic attendances or hospital presentations and/or admissions. Case ascertainment was retrospective based on administrative coding. We acknowledge that this may introduce TIA mimics into our counts, but this was allowed given that our aim was to measure service activity despite the actual final diagnosis. The primary outcome measure was the monthly count of TIA, ischemic stroke, thrombolysis, and EVT occurrences (n/month). Time was measured in months. The proportion of reperfusion candidates was estimated as thrombolysis count or endovascular clot retrieval count as numerator, and ischemic stroke count as denominator.

### 2.4 Statistical analysis

Statistical analysis was performed in R (Statistical Programming Language, version 4.2.1) in two stages – (1) descriptive statistics, and (2) interrupted time series analysis (ITS). Firstly, comparing the pre-COVID and intra-COVID period: we used the Mann–Whitney U test (17) to compare mean monthly rates, and test of proportions compared percentage of stroke requiring IVT or EVT.

Secondly, Interrupted time series analysis was performed using the fpp3 package (18) to calculate β (absolute change in presentation rate), standard error, *t* value, and *p-*value using a previously published method (19). Interrupted time series analysis is a segmented regression approach to test an effect of an intervention on a time-dependent outcome using a defined impact model. A linear model was initially fitted, but if any residual autocorrelation existed [i.e., a linear relationship between lagged variables tested by portmanteau (Box Pierce) testing (18, 20)], autoregressive integrated moving average (ARIMA) errors were introduced (18). Therefore, time varying confounders were considered, and accounted for using time series regression techniques (21). ARIMA models were fitted with the Hyndman-Khandakar algorithm (22), which is a step-wise method of fitting time series data by (1) using unit root testing for stationarity (23), (2) applying a process called differencing if stationarity exists, and (3) automating model fitting based on the lowest possible AICc (corrected Akaike Information Criterion) value (22). Relative change from baseline was calculated as absolute change divided by the average baseline count per month.

## 3 Results

### 3.1 Participants

A total of 6,231 TIA and 13,191 ischemic stroke presentations were included. Of the ischemic strokes, there were 1,664 (12.6%) instances of IVT and 2,145 (16.3%) instances of EVT. More than half of the study participants were from Australia (52.1%), followed by Canada (35.0%), Italy (7.6%) and England (5.4%).

### 3.2 Descriptive data

**Figure 1:**
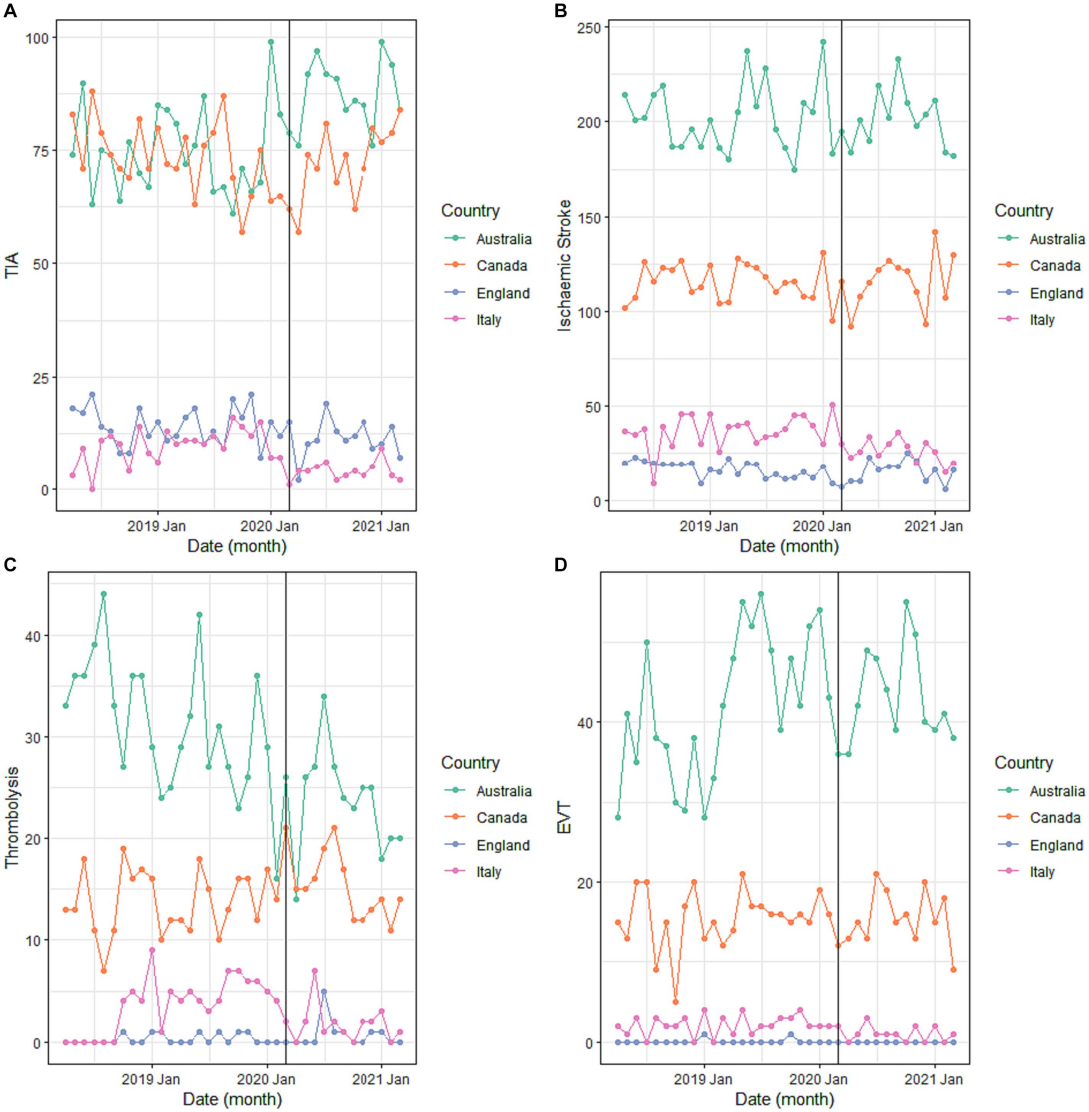
Total monthly counts of **(A)** TIA, **(B)** ischemic stroke, **(C)** EVT, and **(D)** thrombolysis activity from April 2018 to March 2021 by country.

Monthly counts of TIA and ischemic stroke presentations and instances of IVT and EVT are depicted in Figure 1 by country. The results of the univariable analysis are described in Table 2 and summarized next. When comparing the average monthly counts (n/month) for the pre-COVID and intra-COVID period, there was a higher rate of TIA presentations intra-COVID (171–176, *p* = 0.19), lower rate of ischemic stroke presentations (370 to 360, *p* = 0.36), lower rate of monthly IVT counts (49–41, *p* < 0.05), and higher rate of monthly EVT counts (59–60, *p* = 0.80). Only the change in IVT counts was statistically significant. Percentage of ischemic strokes requiring IVT decreased overall from 13.2 to 11.4% (*p* < 0.05). Percentage of ischemic strokes requiring EVT increased overall but this was not significant (16.0–16.7, *p* = 0.33).

**Table 2:** Overall activity of the study population.

			TIA			IS			IVT			EVT			IVT/IS			EVT/IS		
Service	Country	Proportion of total cohort	Before	During	p	Before	During	p	Before	During	p	Before	During	p	Before	During	P	Before	During	P
Monash Medical Center (1)	Australia	15.3%	18	25	<0.05	60	66	0.15	8	5	<0.05	16	16	0.89	12.8	7.0	<0.0001	27.0	24.1	0.15
Royal North Shore (10)	Australia	10.2%	12	13	0.53	42	44	0.72	9	9	0.59	6	9	<0.01	20.8	21.0	0.98	15.1	20.4	<0.05
Royal Adelaide (11)	Australia	26.6%	45	50	0.06	100	92	0.07	15	10	<0.01	19	19	0.84	14.5	10.6	<0.05	19.3	20.3	0.48
John Radcliffe Hospital/OXVASC (2)	England	5.4%	14	11	0.84	16	16	0.84	0	1	0.39	0	0	0.33	1.8	4.8	0.08	0.5	0.0	0.81
Foothills Medical Center (12)	Canada	35.0%	73*	73*	0.94	115	116	0.96	14	15	0.53	15	16	0.97	12.2	12.9	0.56	13.3	13.5	0.92
Bologna (13)	Italy	7.6%	9	4	<0.001	37	26	<0.001	4	2	0.06	2	1	<0.01	9.7	6.7	0.13	5.8	3.8	0.23
Total			171	176	0.19	370	360	0.36	49	41	<0.05	59	60	0.80	13.2	11.4	<0.05	16.0	16.7	0.33

Counts reported as n/month, and proportions as %. Before, baseline pre-COVID period (April 2018 to March 2020). During, intra-COVID period (April 2020 to March 2021). IS, ischemic stroke; TIA, transient ischemic attack; IVT, intravenous thrombolysis; EVT, endovascular thrombectomy; OXVASC, Oxford Vascular Study. *All emergency department visits for a final diagnosis of TIA at all Calgary hospitals, including the subset that were ultimately admitted.

### 3.3 Interrupted time series analysis

**Figure 2:**
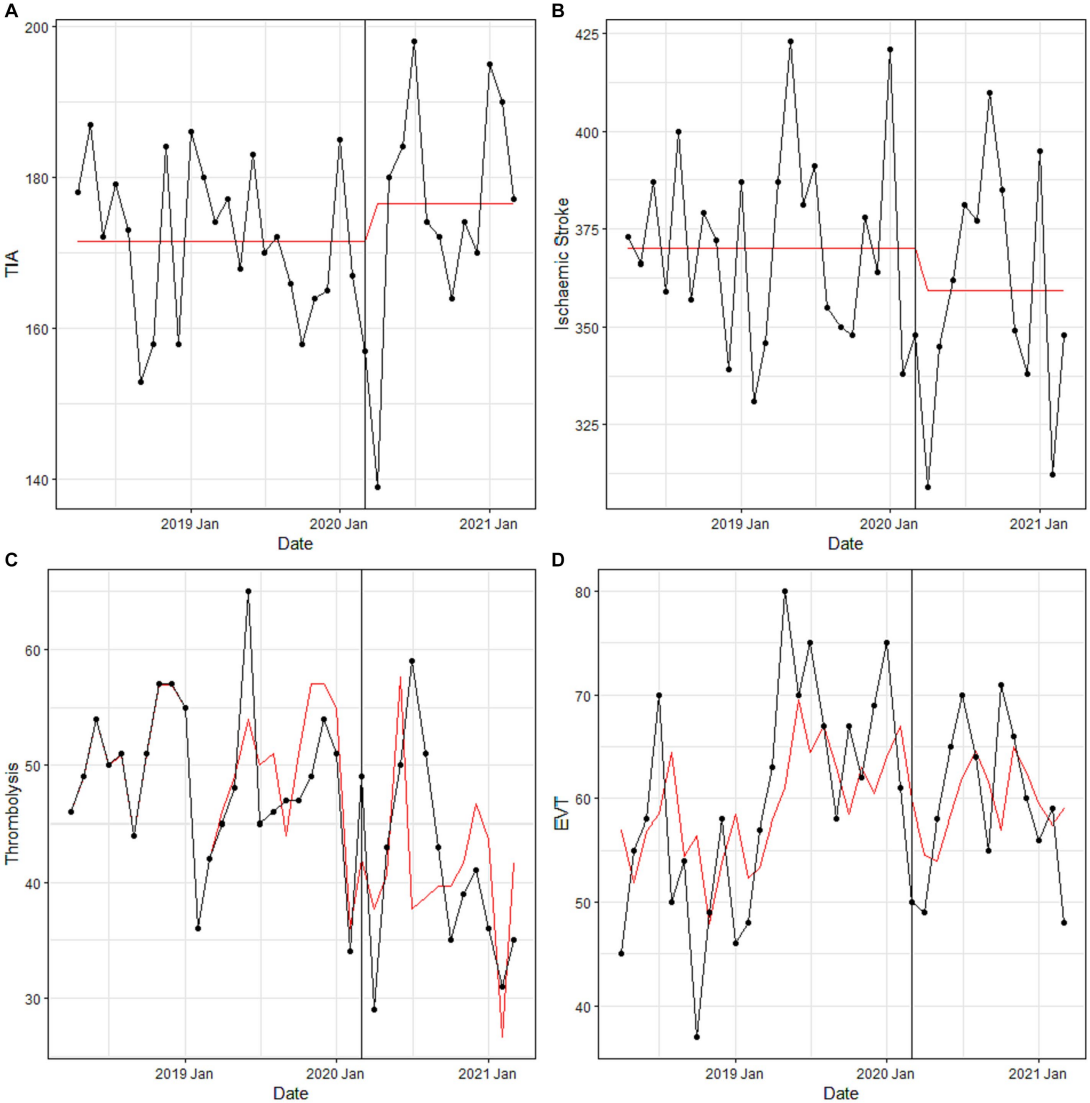
Pooled monthly counts of **(A)** TIA, **(B)** ischemic stroke, **(C)** EVT, and **(D)** thrombolysis activity from April 2018 to March 2021. Black, actual data; Red, fitted model.

Results of the ITS and the calculated relative change from baseline are presented in Table 3 and summarized next. During the pandemic, TIA presentations remained the same (2.9, 95% CI –1.8 to 7.6, *p* = 0.24), as did ischemic stroke (2.9, 95% CI –7.8 to 1.9, *p* = 0.25), IVT (−14.3, 95% CI –36. to 6.1, *p* < 0.01), and EVT (0, 95% CI –16.9 to 16.9, *p* = 0.98) counts. Linear, ARIMA, and seasonal ARIMA models were needed to fit the data to adjust for time varying confounders such as seasonality and autocorrelation, and the exact model used for each of the 24 analyses is listed in Table 3. Visualization of the pooled data and associated fitted models are described in Figure 2.

**Table 3:** Changes in presentation and reperfusion trends during the COVID-19 pandemic including results of the interrupted time series analysis.

	Interrupted time series analysis results					Baseline pre-COVID (n/month)	Relative change from baseline - % (95% CI)
	β (absolute change in n/month)	SE	*t*	*p*	Fitted model		
**TIA**							
Australia	13 (9, 17)	2	5.89	<0.00001	LM + ARIMA (0,0,0)(1,1,0)	75	17.3 (12.0, 22.7)
England	-3 (−6, 0)	1	−2.13	<0.05	LM	14	−21.4 (−42.9, 0)
Canada	0 (−5, 6)	3	0.08	0.94	LM	73	0 (−6.8, 8.2)
Italy	−5 (−8, −3)	1	−4.14	<0.001	LM	9	−55.6 (−88.9, 33.3)
Total	5 (−3, 13)	4	1.19	0.24	LM + ARIMA (0,0,0)	171	2.9 (−1.8, 7.6)
**Ischemic stroke**							
Australia	0 (−12, 11)	6	−0.06	0.95	LM	202	0 (−5.9, 5.4)
England	0 (−4, 4)	2	−0.06	0.95	LM + ARIMA (1,0,0)	16	0 (−25.0, 25.0)
Canada	0 (−7, 8)	4	0.10	0.92	LM	115	0 (−6.1, 7.0)
Italy	−11 (−16, −5)	3	−3.80	<0.001	LM	37	−29.7 (−43.2, −13.5)
Total	−11 (−29, 7)	9	−1.16	0.25	LM + ARIMA (0,0,0)	370	2.9 (−7.8, 1.9)
**IVT**							
Australia	−9 (−23, 5)	7	−1.25	0.22	LM + ARIMA (1,1,0)(1,1,0) [12]	32	−28.1 (−71.9, 15.6)
England	0 (0, 1)	0	1.49	0.14	LM	0	NA
Canada	0 (−2, 3)	1	0.33	0.74	LM + ARIMA (0,0,1)	14	0 (−14.3, 0)
Italy	−2 (−4, 1)	1	−1.49	0.15	LM + ARIMA (1,0,0)	4	−50 (−100.0, 0.0)
Total	−7 (−18, 3)	2	−3.48	<0.01	LM + ARIMA (0,0,0)(0,1,0) [12]	49	−14.3 (−36.7, 6.1)
**EVT**							
Australia	1 (−7, 10)	4	0.34	0.73	LM + ARIMA (1,0,0)	41	2.4 (−17.1, 24.4)
England	0 (0, 0)	0	−1.04	0.30	LM	0	NA
Canada	0 (−2, 3)	1	0.20	0.84	LM	15	0 (−13.3, 20.0)
Italy	−1 (−2, −1)	0	−5.09	<0.0001	LM + ARIMA (1,0,0)	2	−50 (−100.0, −50.0)
Total	0 (−10, 10)	5	0.02	0.98	LM + ARIMA (1,0,0)	59	0 (−16.9. 16.9)

SE, standard error; LM, linear model; ARIMA, autoregressive integrated moving average; TIA, transient ischemic attack; EVT, endovascular thrombectomy.

Results of the ITS by country are also presented in Table 3 and summarized next. The centers from Australia reported an increase in TIA presentations of 17.3% (95% CI 12.0 to 22.7, *p* < 0.00001), while the center from UK (−21.4, 95% CI -42.9 to 0, *p* < 0.05) and Italy (−55.6, 95% CI –88.9 to 33.3, *p* < 0.001) reported decreases, leading to an overall non-significant change in rate. The fall in ischemic stroke counts appeared to be largely due to reduction of −29.7% (−43.2 to −13.5, *p* < 0.001) in the center from Italy. Regarding reperfusion, only EVT counts (−50, 95% CI –100.0 to −50.0, *p* < 0.0001) from Bologna changed significantly. Results of the ITS by center are presented in Supplementary Table S1.

## 4 Discussion

The major finding of this study was that TIA volumes remained constant even during the pandemic, and this remained true for monthly ischemic stroke, IVT, and EVT counts. This suggests that the pandemic has not had enduring effect on TIA clinic activity despite early reports of significant reductions in TIA incidence and admissions. Secondarily however, the disproportionate decrease in IVT during this period suggests that patients may be presenting outside the IVT window during the pandemic – delays in seeking treatment in this group could be the target for public health intervention.

### 4.1 TIA presentations

Our study demonstrated that TIA clinic volume has remained constant over 3 years, inclusive of a 1-year intra-COVID period, and this provides evidence against initial concerns regarding patients with transient symptoms not presenting for healthcare. Previous authors have hypothesized about this reluctance (6, 24, 25), but these were made during an earlier phase of the COVID-19 pandemic. Our study adds value to these initial reports as it includes (1) an extended period of observation and (2) includes hospitals from four different countries to allow initial investigation of regional differences. It is important to note that the experience within our group was varied, with the three centers from Australia seeing an increase in TIA presentations of 17.3% as opposed to the decrease of 21.4% at the center from UK and 55.6% at the center from Italy. Regional differences may explain this contrast, including differences in COVID-19 incidence and mortality, governmental policy, public health awareness and campaigns, and cultural differences in health-seeking behavior, however these were not captured by this study. Similar single-country reports however were consistent with our data, with three hyperacute stroke centers in the UK describing an immediate reduction in TIA referrals by 28% after the first UK COVID death on 9th March 2020 (26) and a tertiary stroke care center in Italy describing a 51% decrease in TIA incidence when comparing 8th March to 2nd May 2020 to the corresponding period in 2019 (6). In contrast, a center in Hong Kong reported no increase in referrals to TIA clinic in January to March 2020, despite fewer TIA cases being admitted to their hospital when compared to the same period in 2019 (8). Together, the existing evidence combined with our results demonstrates region-specific differences in TIA activity in response to COVID-19 and highlights the complexity in a patient’s decision to seek care for transient and/or minor symptoms. Nevertheless, the overall result of constant TIA clinic activity throughout the pandemic suggests that the overall impact of COVID-19 may have only been temporary, and that patients are still accessing care via rapid TIA clinics. Overall, we believe that the previously documented adaptations of each clinic in response to the pandemic (9) have contributed to meeting service demand.

### 4.2 Ischemic stroke presentations and reperfusion trends

Secondarily, our study demonstrated reductions in ischemic stroke presentations and a disproportionate decrease in IVT during the pandemic period, and this contrasts with TIA clinic activity described earlier. This group may be a separate demographic to TIA patients, perhaps more advanced in age, and perhaps more reluctant to seek care that the TIA group. The data reverses the pattern of larger reductions in TIA incidence than ischemic stroke incidence in both primary care (5) and hospital (6), and further highlights this group of patients as a distinct subgroup. Nevertheless, the reductions in ischemic stroke presentations remain consistent with published studies, with papers reporting reductions in stroke codes (7, 24, 27), stroke presentations (28, 29), new stroke diagnoses (25), stroke admissions (7, 26, 30–34), and stroke discharges (35). Similarly, our reduction in IV thrombolysis numbers was consistent with data from France (27), China (30), and Iran (32), but data from Japan (34) and US (36) did not report changes in IV thrombolysis numbers. This could be because patients were presenting later and outside the IVT window, leading to our observed 14.3% reduction in thrombolysis counts. It is not clear whether patients may be presenting elsewhere or whether they are simply presenting later, but a study of 1,262 general practices in Germany noted a 10% reduction in stroke incidence in April to June 2020 when compared to the corresponding period in 2019, suggesting that patients may not be presenting to their general practitioners either (5). Lastly, we did not observe any significant change in endovascular clot retrieval activity, similar to the Japan (34) and US (36) studies, but note that the centers from France (27) and China (30) did experience significant reductions. In conclusion, delays in seeking treatment in what appears to be a separate subgroup of patient behavior from those with transient and/or minor symptoms could be a valuable target for public health intervention during periods of public concern, especially pandemics.

### 4.3 Interrupted time series

Various methods have been used to describe service-level changes as a result of COVID-19, including cohort comparisons to the corresponding period in 2019 (8, 27, 29, 30, 35, 37), comparisons to monthly averages over years (28), or as before and after *t*-testing (25). These previous approaches make certain *a priori* assumptions about the timing of the impact of an event and allow only two groups: ‘before’ and ‘after.’ However, interrupted time series analysis is a useful study design to evaluate the effectiveness of population-level interventions because (1) there is flexibility in modelling the impact (e.g., level and/or slope change, temporary or permanent change) and (2) consideration of time varying confounders such as seasonal trends and adjustment for autocorrelation of data (21). Consecutive observations over time can tend to be more similar to closer months than further months, a phenomenon known as autocorrelation, and this violates the assumption of non-ITS regression models that observations need to be independent (21). Not many studies choose this advanced statistical approach to analyze the impact of COVID on service-level data, which highlights the methodological strength of our analysis (19, 26, 31–34, 38).

### 4.4 Strengths and limitations

The follow-up duration was longer than most existing studies, providing a more longitudinal view of pandemic health service impact beyond the initial acute phase. Sampling from multiple countries is a strength, but variations in COVID-19 community levels, durations of Governmental lockdown, and presence of public awareness campaigns remain that may influence results from each center. Furthermore, the proportion of mimics was not recorded in the data, and it is known that up to 38% of TIA clinic attendees are ultimately diagnosed as mimics (1). Lastly, more than half of the study population was from centers in Australia, which is not representative of rapid TIA clinics in general.

### 4.5 Generalizability

These findings are expected to be valid in stroke centers that offer rapid TIA clinics, as well as systemic and/or mechanical reperfusion. Hospitals that intend to apply these findings to current and future planning could use this data as an overall direction of effect but would benefit from an individualized time series prediction of their own presentation numbers.

## 5 Conclusion

TIA clinic volumes remained constant even during the pandemic, as did monthly ischemic stroke, IVT, and EVT counts. TIA clinic and stroke service volume for these centers therefore appears to have remained constant. However, the disproportionate decrease in IVT suggests that patients may be presenting outside the IVT window during the pandemic. Delays in seeking treatment in this group could be the target for public health intervention.

## Data availability statement

The raw data supporting the conclusions of this article will be made available by the authors, without undue reservation.

## Ethics statement

The studies involving humans were approved by Monash Health Human Research Ethics Committee. The studies were conducted in accordance with the local legislation and institutional requirements. Written informed consent for participation was not required from the participants or the participants’ legal guardians/next of kin in accordance with the national legislation and institutional requirements.

## Author contributions

AL: Conceptualization, Data curation, Formal analysis, Investigation, Methodology, Software, Visualization, Writing – original draft, Writing – review & editing. PR: Supervision, Writing – review & editing, Conceptualization, Data curation, Methodology. LL: Conceptualization, Data curation, Writing – review & editing, Resources. SC: Conceptualization, Data curation, Resources, Writing – review & editing. MH: Conceptualization, Data curation, Resources, Supervision, Writing – review & editing. MG: Conceptualization, Data curation, Resources, Writing – review & editing. VB: Conceptualization, Data curation, Resources, Writing – review & editing. FR: Conceptualization, Data curation, Resources, Writing – review & editing. TK: Conceptualization, Data curation, Resources, Supervision, Writing – review & editing. RC-F: Data curation, Writing – review & editing. MK: Conceptualization, Data curation, Resources, Supervision, Writing – review & editing. MW: Data curation, Writing – review & editing. SS: Conceptualization, Writing – review & editing. HM: Conceptualization, Project administration, Resources, Supervision, Writing – review & editing. TP: Conceptualization, Data curation, Investigation, Methodology, Software, Supervision, Writing – original draft, Writing – review & editing.
