# Supplementary material for: Rapid outpatient transient ischemic attack clinic and stroke service activity during the SARS-CoV-2 pandemic: a multicenter time series analysis
Source: Front Neurol. 2024 Feb 7;15:1351769. doi: 10.3389/fneur.2024.1351769 (PMC10879819; doi:10.3389/fneur.2024.1351769)
Supplement: Supplementary file 1 [file Table_1.docx]

Supplementary Table S1. Changes in presentation and reperfusion trends during the COVID-19 pandemic including results of the interrupted time series analysis by center and years since publication.

|  |  | Interrupted time series analysis results | | | | | Baseline pre-COVID (n/month) | Relative change from baseline - % (95% CI) |
| --- | --- | --- | --- | --- | --- | --- | --- | --- |
|  | Years since publication | β (absolute change in n/month) | SE | t | p | Fitted model |  |  |
| **TIA** |  |  |  |  |  |  |  |  |
| John Radcliffe Hospital / OXVASC | 14 | -3 (-6, 0) | 1 | -2.13 | <0.05 | LM | 14 | -21.4 (-42.9, 0) |
| Monash Medical Center | 9 | 15 (6, 23) | 4 | 3.49 | <0.01 | LM + ARIMA (0,0,0)(0,1,1)[12] | 18 | 83.3 (33.3, 127.8) |
| Foothills Medical Center | 9 | 0 (-5, 6) | 3 | 0.08 | 0.94 | LM | 73 | 0 (-6.8, 8.2) |
| Bologna | 6 | -5 (-8, -3) | 1 | -4.14 | <0.001 | LM | 9 | -55.6 (-88.9, 33.3) |
| Royal North Shore | 5 | 0 (-1, 3) | 1 | 0.82 | 0.42 | LM + ARIMA (0,0,0) | 12 | 0 (-8.3, 25.0) |
| Royal Adelaide | 3 | 6 (2, 10) | 2 | 3.13 | <0.01 | LM + ARIMA (0,0,0)(1,1,0)[12] | 45 | 13.3 (4.4, 22.2) |
| Total |  | 5 (-3, 13) | 4 | 1.19 | 0.24 | LM + ARIMA (0,0,0) | 171 | 2.9 (-1.8, 7.6) |
